# In-Vitro Generation of T‑Cell Clones to Difluorophenylglyoxal: Implications for Understanding the Immunogenicity of MK-3207

**DOI:** 10.1021/acs.chemrestox.6c00294

**Published:** 2026-07-30

**Authors:** Paul Thomson, Andrew Gibson, Jackie Shang, Xiaoli Meng, Eleanor Saville, Sophie Grice, Megan Ford, Yu Feng, Kara Pearson, Michael Oropallo, Raymond Gonzalez, James Monroe, Dean Naisbitt

**Affiliations:** ‡ Department Pharmacology and Therapeutics, University of Liverpool, Liverpool L693GE, U.K.; † Institute for Immunology and Infectious Diseases, Murdoch University, Murdoch WA 6150, Australia; § Investigative Toxicology, Nonclinical Drug Safety, Merck & Co., West Point, Pennsylvania 19486, United States

## Abstract

MK-3207 was withdrawn from development because of drug-induced liver injury. Metabolic studies identified bioactivation to a protein-reactive 3,5-difluorophenylglyoxal (DFPG) intermediate. Although phenylglyoxal metabolites readily form protein adducts, their immunogenic potential has been poorly understood. We compared the immunogenicity of three DFPG isomers (3,5-, 2,4-, and 3,4-DFPG) using T-cell clones generated from treated healthy donor PBMCs. Reactive clones were detected against isomers with 3,5-DFPG inducing the strongest responses. Activation required antigen processing, and no cross-reactivity with MK-3207 was observed. These findings demonstrate the immunogenicity of 3,5-DFPG and show that structural differences among phenylglyoxal isomers influence T cell activation.

## Graphical abstract

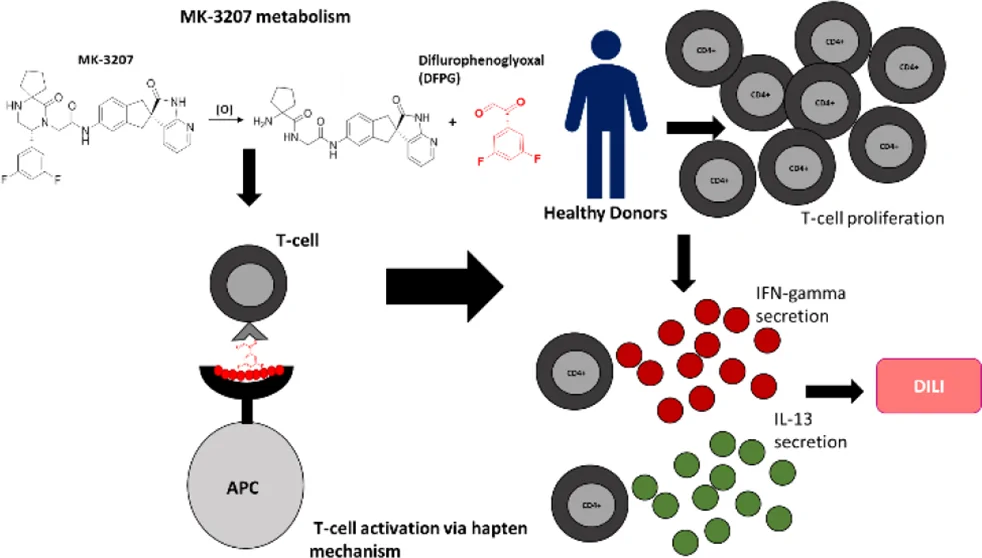

MK-3207 is a calcitonin gene-related peptide (CGRP) antagonist developed for the treatment of migraine without the vasoconstrictive liabilities associated with first-line triptan therapy. However, development of MK-3207 was discontinued following the emergence of delayed-onset liver injury in patients, consistent with an immune-mediated mechanism.^1,2^ Despite extensive repeat-dose toxicity studies in rodents and nonrodents at high exposures, liver toxicity signals comparable to those observed clinically were not detected.

We have previously demonstrated the presence of drug-responsive T-cells from patients with drug-induced liver injury alongside skin allergy, thus providing direct evidence for their involvement in disease pathogenesis.^2–13^ Furthermore, histological examination of inflamed liver from patients exposed to sulfasalazine, ibuprofen, and atabecestat revealed infiltration of mediator-secreting T-lymphocytes.^12–14^ Given the occurrence of adverse hepatic reactions following exposure to MK-3207, we sought to determine whether similar drug-reactive T-cell clones could be detected.

Metabolite identification studies revealed the formation of a 3,5-difluorophenylglyoxal (3,5-DFPG) metabolite of MK-3207 (Scheme 1), the reactivity of which was confirmed in a peptide trapping experiment (Figure 1). Exposure to 3,5-DFPG resulted in the modification of a cysteine residue, forming a DFPG adduct. In addition, the identical modified peptide was detected at lower abundance following incubation of MK-3207 with CYP3A4 and NADPH to generate 3,5-DFPG in situ. The identical retention and MS/MS fragmentation patterns of the DFPG-modified peptide were observed under both incubation conditions. Fragment ions highlighted by an asterisk (*) contain the DFPG adduct on the cysteine residue (Figure 1).

**Figure 1:**
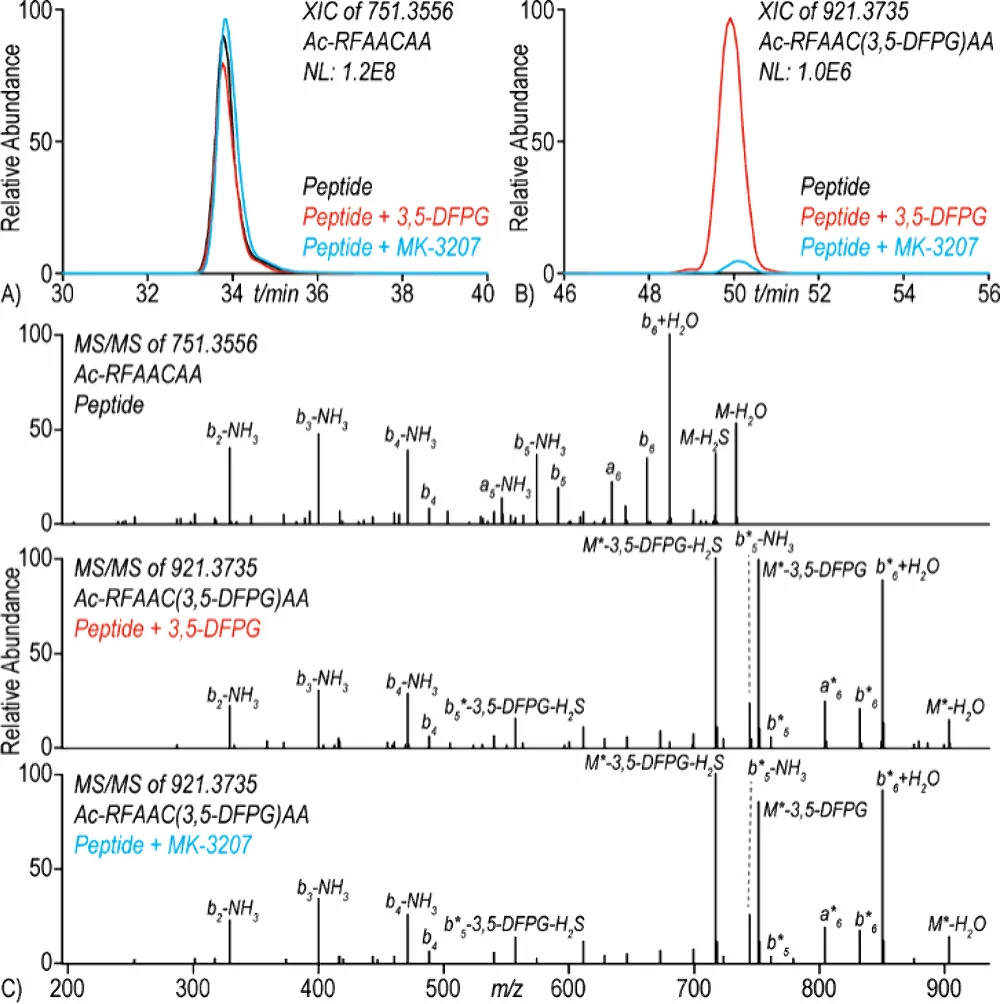
Peptide trapping experiment confirmed adduct formation following incubation with 3,5-DFPG and MK-3207. (A) Unmodified peptide Ac-RFAACAA was detected in all conditions including control (Peptide alone), 3,5-DFPG incubation (Peptide +3,5-DFPG) and MK-3207 incubation (Peptide + MK-3207+ CYP3A4 + NADPH). (B) 3,5-DFPG-modified peptide was detected following incubation with both 3,5-DFPG and MK-3207. (C) MS/MS analysis confirmed formation on a 3,5-DFPG adduct on the cysteine residue, with identical fragmentation patterns in both 3,5-DFPG and MK-3207 incubation conditions. Fragment ions with asterisk (*) contain a 3,5-DFPG-modified cysteine residue.

Phenylglyoxal compounds are known to covalently modify specific amino acid residues in human serum proteins.^15^ Indeed, three structural isomers, differing in the positions of the fluorine substituents on the benzine ring (2,4-; 3,4-; 3,5-) Propensity of DFPG analogues to modify human serum proteins and its associated antigenicity (Scheme 1, were found to modify amino acids such as arginine, lysine, and cysteine.^15a^ While all of these isomers can form drug adducts, their individual immunogenicity has not been determined.

In this study, the isomers of DFPG were used to investigate their propensity to activate T-cells from seven healthy human donors. PBMCs (1 × 10^6^) were cultured with nontoxic concentrations of each isomer for 14 days, followed by serial dilution and repeated mitogen stimulation using established methods to generate T-cell clones.^3,4^ To assess compound specificity, T-cell clones were cultured in duplicate (5 × 10^4^ per well; 96-well plate; total volume 200 μL) with autologous Epstein–Barr virus (EBV)-transformed B-cells (1 × 10^4^ per well) in either R9 media (negative control) or test compound (MK-3207, 2.5 μM; DFPG structural isomers, 40 μM) for 48 h. Subsequently, [^3^H]-thymidine was added and cultures were incubated for an additional 16 h before cellular proliferation was quantified by scintillation counting. Once expanded into multiple wells, dose-dependent (DFPG isomers, 40–300 μM) T-cell responses were characterized using proliferation or ELIspot assays as readouts. To understand the pathway of T-cell activation with DFPG, a trio of assays were performed. EBV-transformed B-cells were (i) fixed using glutaraldehyde to prevent antigen uptake and processing, (ii) pulsed with DFPG for periods ranging from 10 min to 24 h, followed by washing to remove free compound prior to coculture with T-cells in the absence of additional soluble drug antigen, and (iii) pretreated with MHC class I and II blocking antibodies alongside their respective isotype controls.

**Scheme 1:**
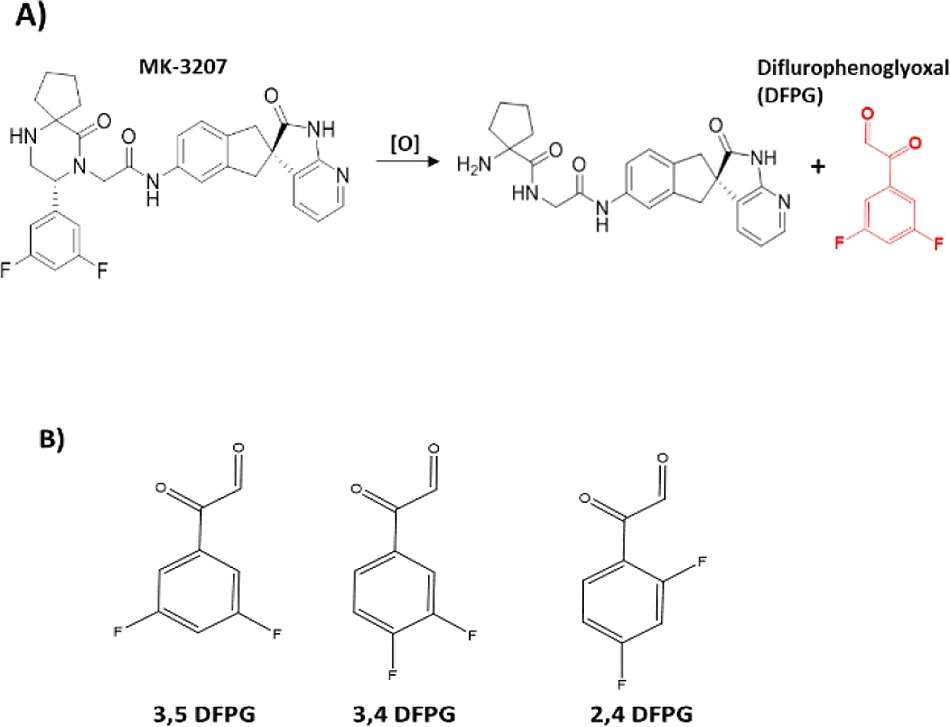
MK-3207 Metabolism Yielding DFPG and DFPG Isomer Structures

Twenty-eight T-cell clones derived from seven compound-naïve donors displayed proliferative responses to 3,5-DFPG; (Figure 2A; results from three representative donors are shown). A subfraction of four 3,5-DFPG-responsive clones displaying dose-dependent proliferative responses and cytokine release (Figure 2B and D) were expanded for further mechanistic characterization. These T-cell clones showed no cross-reactivity with MK-3207 indicating a conserved and highly specific T-cell reactivity profile (Figure 2C). Although 3,4- and 2,4-DFPG clones were detected during initial testing (stimulation index 1.5–3), these responses were not reproduced in dose–response studies following clonal expansion. T-cell cloning to respective isomers in seven healthy donors is summarized in Table 1.

**Figure 2:**
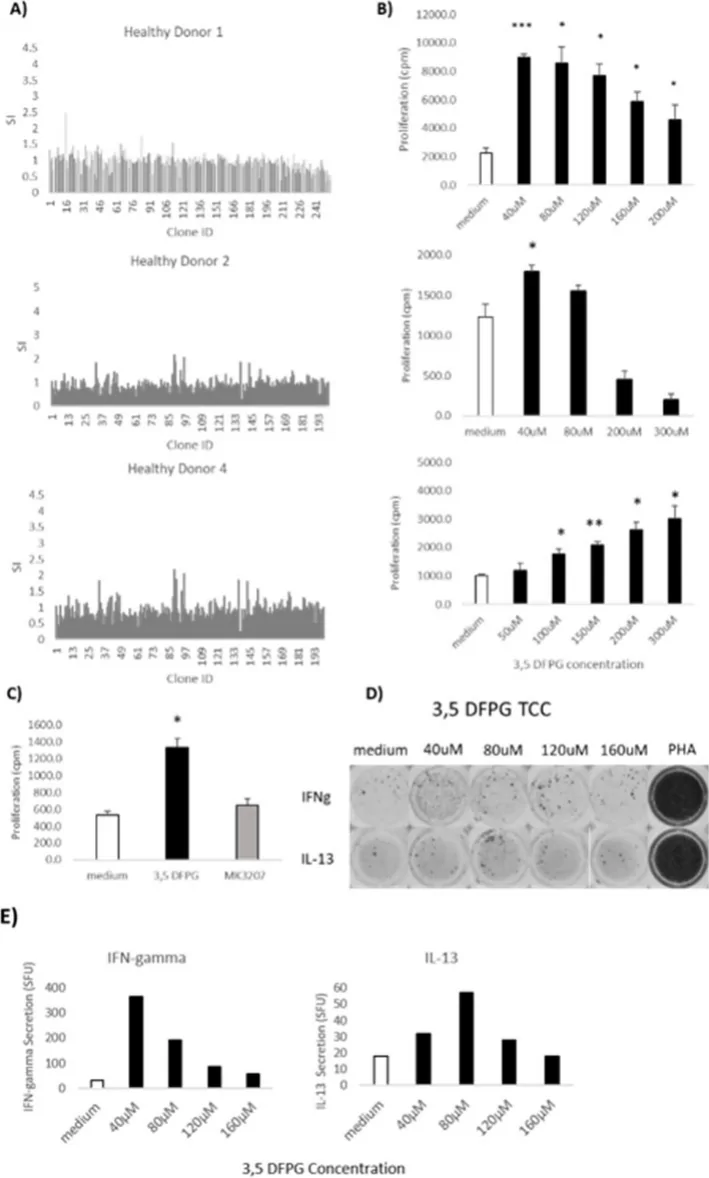
Activation of T-cell clones from healthy human donors with 3,5-DFPG. (A) Initial specificity testing of T-cell clones derived from healthy donors to 3,5-DFPG. Clones (5 × 10^4^/50 μL) were incubated with autologous EBV-transformed B-cells (1 × 10^4^/50 μL) in the presence and absence of 3,5-DFPG (40 μM) in a U-bottomed 96 well microplate. Cells were incubated for 48 h (37 °C; 5% CO_2_) and [3H] thymidine was added for the last 16 h. Proliferative responses were assessed using scintillation counting. Clones with a stimulation index of ≥ 1.5 were selected, expanded and subjected to dose–response studies. T-cell clones (5 × 10^4^/well; 96-well plate) were cultured for 48 h with autologous EBV-transformed B-cells (1 × 10^4^/well) and 3,5-DFPG (40–200 μM), or MK-3207 in 96-well U-bottomed plates for (B) proliferation analysis of dose response and (C) cross-reactivity or (D) analysis of cytokine secretion on cytokine-specific capture antibody precoated ELISpot plates. For ELISpot analysis, plates were developed according to the manufacturer’s instructions for the major Th1 (IFN-γ) and Th2 (IL-13) cytokines. (E) cytokine secretion quantified as spot forming units (SFU), doubling of background SFU by ≥10 was classified as positive. Data displays the average of triplicate cultures + SD. Asterisks denote statistical significance [(*) *p* < 0.05, (**) *p* < 0.005].

**Table 1:** T-Cell Cloning from Seven Healthy Donors to DFPG Isomers^a^

Healthy Donor	2,4 DFPG	3,4 DFPG	3,5 DFPG
1	0/231	0/280	4/254
2	0/136	0/208	8/200
3	N/A	0/306	0/63
4	N/A	N/A	11/80
5	0/92	0/99	0/75
6	0/160	0/160	1/104
7	N/A	N/A	4/408

a Number of drug responsive T-cell clones/number of clones tested. N/A = T-cell cloning not conducted due to cell number limitations.

The 3,5-DFPG-induced proliferative response was abrogated upon inclusion of an MHC class II but not MHC class I blocking antibody. (Figure 3A). This is consistent with the CD4^+^ phenotyping of clones using flow cytometry. T-cell clones were activated in the presence of 3,5-DFPG and autologous antigen-presenting cells; however, similar proliferative responses were not observed when antigen-presenting cells were fixed with glutaraldehyde to block antigen processing (Figure 3B). Furthermore, stimulation of T-cells using antigen-presenting cells pulsed with 3,5-DFPG for various durations indicated that protein adduct formation is required for T-cell activation (Figure 3C).

**Figure 3:**
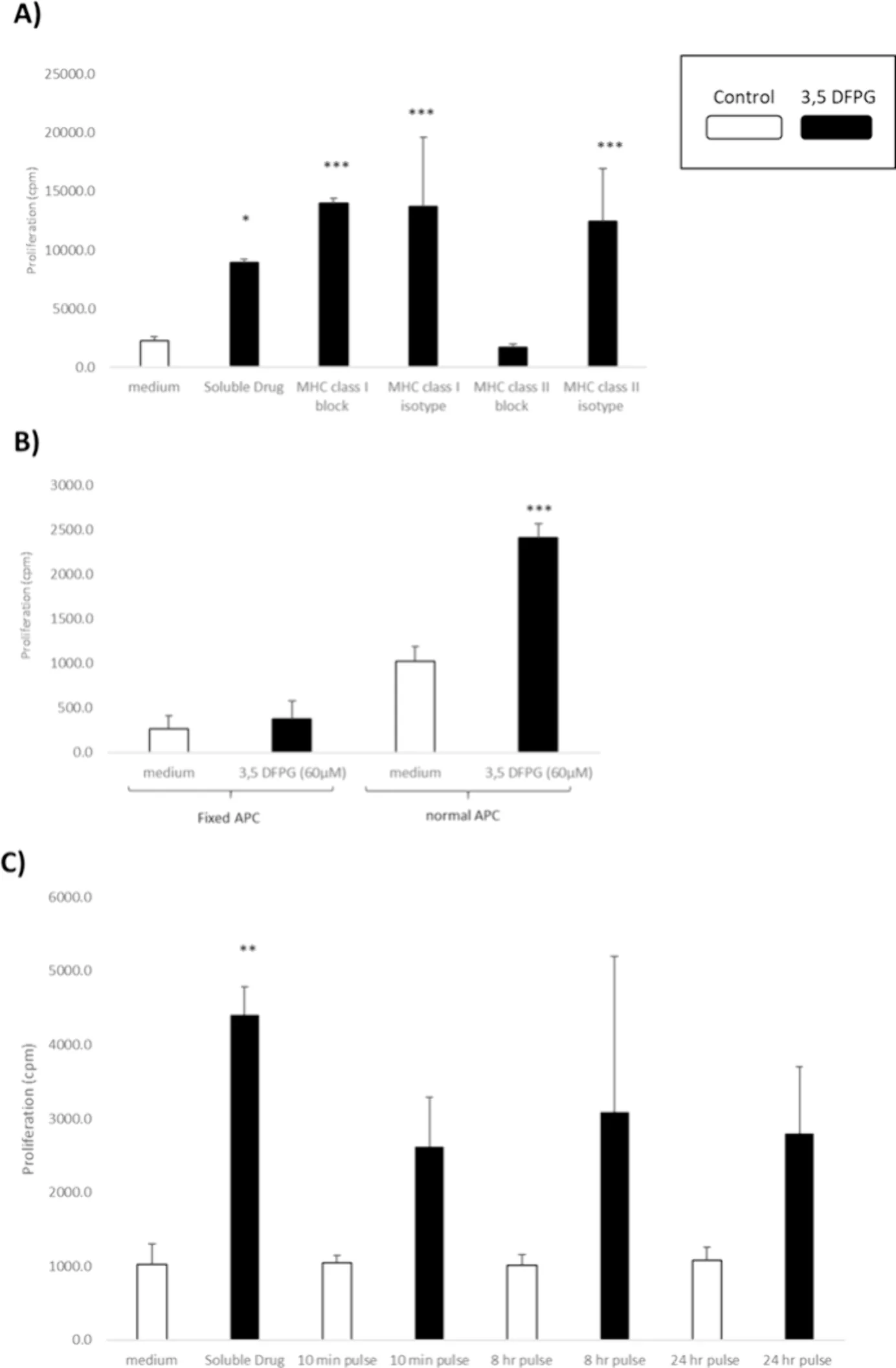
3,5-DFPG-responsive CD4+ T-cell clones are activated by an antigen processing-dependent and MHC class II-restricted pathway. Clones (5 × 10^4^/well; 96-well plate) were cultured for 48 h with autologous irradiated EBV-transformed B-cells (1 × 10^4^/well) in 96 well U-bottomed plates with (A) MHC class I or II blocking antibodies, alongside their respective isotype controls, or (B) glutaraldehyde fixed antigen presenting cells before addition of 3,5 DFPG (40 μM) and culture for 48 h. (C) Autologous EBV-transformed B-cells were cultured with 3,5 DFPG (40 μM) for time periods ranging between 10 min to 24 h before several washes removed free drug and pulsed APCs were then plated (1 × 10^4^/well) with the T-cell clone (5 × 10^4^/well; 96-well plate) in the absence of free compound for 48 h. [^3^H] thymidine was then incorporated and cells cultured for a further 16 h before harvesting for analysis of cellular proliferation. Data displays the average of triplicate cultures + SD. Asterisks denote statistical significance [(*) *p* < 0.05, (**) *p* < 0.005, (***) *p* ≤ 0.001].

The lack of clearly defined risk factors and predictive preclinical safety models for identifying individuals at risk of immune-mediated drug-induced liver injury represents a major challenge for clinicians, patients, and the pharmaceutical industry. Drug-specific T-cells that drive these reactions have been isolated from hypersensitive patients, and their functional characterization has provided insight into the antigenic determinants driving memory T-cell responses. Preclinical immunogenicity assessment requires screening of parent compounds and immunologically relevant metabolites using naïve T-cells derived from drug-inexperienced human donors. The role of CD4+ T-cells in drug induced liver injury has previously been demonstrated in patients.^16^ CD4+ T-cells have been known to enhance the activity of CD8+ T-cells as well as mediate the intensity of and duration of immune responses, while also imposing direct toxicity on tissues through the secretion of cytotoxic mediators.^17,18^ This study highlights the heightened immunogenicity of the protein-reactive 3,5-DFPG metabolite over the parent compound MK-3207. Notably, 3,5-DFPG activated T-cells more robustly than other DFPG structural isomers. This observation is consistent with reports that fluoroquinolones (e.g., temafloxacin and trovafloxacin) that contain 2,4-DFPG moieties are associated with similar immunotoxicity profiles, but at a significantly lower frequency than observed for MK-3207.^19^ This implies that the overall immunogenicity and immunotoxicity of DFPG metabolites is influenced by the positions of the fluorine atoms, providing a potentially novel structural–activity relationship in drug-induced immune activation.^20^ 3,5-DFPG-responsive CD4+ T-cell clones were generated across multiple compound naïve donors and were stimulated to proliferate and secrete pro-inflammatory cytokine IFN-γ, in a dose dependent manner. Clones did not cross-react with MK-3207 indicating a conserved specificity. Mechanistic studies conducted with the 3,5-DFPG-responsive CD4+ T-cell clones determined that T-cell activation was MHC class II restricted and dependent on protein adduct formation and subsequent processing to form haptenated peptides. To conclude, these data demonstrate the ability to generate DFPG-responsive T-cells from healthy, compound naïve donors and implicate the reactive 3,5-DFPG metabolite as a key contributor to the immune-mediated liver injury observed in clinical trial participants exposed to MK-3207.

## ABBREVIATIONS

HLA: human leukocyte antigen
PBMC: peripheral blood mononuclear cells
SI: stimulation index
MHC: major histocompatibility complex
